# Placental extracellular vesicles express active dipeptidyl peptidase IV; levels are increased in gestational diabetes mellitus

**DOI:** 10.1080/20013078.2019.1617000

**Published:** 2019-05-23

**Authors:** Neva Kandzija, Wei Zhang, Carolina Motta-Mejia, Vuyane Mhlomi, Jennifer McGowan-Downey, Tim James, Ana Sofia Cerdeira, Dionne Tannetta, Ian Sargent, Christopher W. Redman, Claire C. Bastie, Manu Vatish

**Affiliations:** a Nuffield Department of Women’s and Reproductive Health, University of Oxford, Oxford, UK; b College of Health and Life Sciences, Brunel University London, Uxbridge, UK; c Department of Natural Sciences, University of Bath, Bath, UK; d Department of Clinical Biochemistry, Oxford University Hospitals NHS Trust, Oxford, UK; e Department of Food and Nutritional Sciences, University of Reading, Reading, UK; f Division of Biomedical Sciences, Warwick Medical School, Coventry, UK

**Keywords:** Placental extracellular vesicles, syncytiotrophoblast-derived extracellular vesicles, gestational diabetes mellitus, gliptin, pregnancy, dipeptidyl peptidase IV

## Abstract

Gestational diabetes mellitus (GDM) is the most common metabolic disorder in pregnancy and is characterized by insulin resistance and decreased circulating glucagon-like peptide-1 (GLP-1). GDM resolves rapidly after delivery implicating the placenta in the disease. This study examines the biological functions that cause this pathology. The placenta releases syncytiotrophoblast-derived extracellular vesicles (STB-EVs) into the maternal circulation, which is enhanced in GDM. Dipeptidyl peptidase IV (DPPIV) is known to play a role in type 2 diabetes by breaking down GLP-1, which in turn regulates glucose-dependent insulin secretion. STB-EVs from control and GDM women were analysed. We show that normal human placenta releases DPPIV-positive STB-EVs and that they are higher in uterine than paired peripheral blood, confirming placental origin. DPPIV-bound STB-EVs from normal perfused placentae are dose dependently inhibited with vildagliptin. DPPIV-bound STB-EVs from perfused placentae are able to breakdown GLP-1 *in vitro*. STB-EVs from GDM perfused placentae show greater DPPIV activity. Importantly, DPPIV-bound STB-EVs increase eightfold in the circulation of women with GDM. This is the first report of STB-EVs carrying a biologically active molecule that has the potential to regulate maternal insulin secretion.

## Introduction

Gestational diabetes mellitus (GDM) is defined as glucose intolerance resulting in hyperglycaemia, with an onset or first recognition during pregnancy [1]. In 2015, approximately 17.8 million of births were affected by GDM [2], with increasing prevalence directly linked to increasing risk factors (e.g. obesity) in women of reproductive age [3].

GDM causes short- and long-term complications for both mother and foetus. Maternal short-term risks include caesarean section, post-partum haemorrhage and/or preeclampsia, while the foetus is at increased risks of macrosomia, birth trauma and a number of metabolic imbalances including hypoglycaemia, consequently increasing the risk of neonatal unit admission. Moreover, women with GDM are at elevated risk for developing impaired glucose tolerance and type 2 diabetes later in life. It is reported that up to 70% of women with GDM develop type 2 diabetes within 5 years of delivery [4]. Children of women with GDM also have increased lifetime risks of obesity and are more likely to have impaired glucose tolerance and diabetes in childhood and early adulthood [5,6].

Control pregnancy is characterized by a progressive decrease in maternal insulin sensitivity, which facilitates glucose and lipid bioavailability for the foetus [7,8]. As such, hyperinsulinemia with euglycemia is observed in control pregnant women. GDM is characterized by an exacerbation of this physiological insulin resistance state associated with an insufficient insulin secretion, ultimately resulting in hyperglycaemia [9].

The placenta is known to be centrally involved in inducing the insulin resistance seen in this condition and delivering the placenta rapidly improves GDM in the short term, with simultaneous decreases in the requirements for insulin and/or metformin and reduction of glucose levels to normal; yet the molecular mechanisms by which the placenta impacts the maternal insulin function remain unknown [10].

It is now well established that the syncytiotrophoblast monolayer covering the placental chorionic villi releases extracellular vesicles (EVs) into the maternal circulation during pregnancy [11–13]. The placental origin of syncytiotrophoblast-derived extracellular vesicles (STB-EVs) is determined by the expression of the placental alkaline phosphatase (PLAP) present in these EVs [14,15]. EVs are classified as “small EV” (<200 nm; in this manuscript SMALL STB-EV) and “medium/large EVs” (>200 nm; MEDIUM/LARGE STB-EV) according to their size [16]. Importantly, STB-EV shedding from the placenta increases as the pregnancy progresses, and positively correlates with the physiological increase of insulin resistance during pregnancy [13,17]. As such, STB-EVs might allow communication between the placenta and the maternal metabolism. Interestingly, circulating levels of SMALL STB-EVs have been reported to be elevated in GDM [18] and their role in other placenta-associated disorders has been well described [19,20]. This strongly supports the concept that STB-EVs are not simple debris, but can interact with maternal cells.

Therefore, we hypothesized that STB-EVs might carry factors impacting insulin secretion, a hallmark of pregnancy that is altered during the establishment of GDM. For the reasons above, we investigated the presence and biological function of Dipeptidyl peptidase IV (DPPIV). DPPIV is a glycoprotein that rapidly cleaves the *N*-terminal dipeptides of incretin hormones (e.g. glucagon-like peptide 1 (GLP-1)), which are known to increase insulin secretion, and thus regulate glucose homeostasis. It is known that DPPIV is elevated in type 2 diabetes mellitus (T2DM) and DPPIV inhibitors are established as therapeutic agents for the treatment of T2DM [21]. The potential role of placentally derived DPPIV in GDM has not previously been investigated. Our hypothesis was that biologically active DPPIV is shed on STB-EVs and there might be increased circulating levels in GDM.

## Material and methods

### Patients and samples

This project was approved by the Central Oxfordshire Research Ethics Committee C (REFS 07/H0607/74 and 07/H0606/148). All participants gave written informed consent. All participants were pregnant women scheduled for elective caesarean section at 39–40 weeks of gestation. Oral glucose tolerance tests (OGTTs) were performed at 26–28 weeks of gestation to diagnose GDM. Criteria for GDM diagnosis were either a fasting plasma glucose level ≥ .1 mmol/l and/or a glucose level ≥10.1 mmol/l at 1 h and/or ≥8.5 mmol/l at 2 h after a 75 g oral glucose load, and followed the cut-off values established by guidelines from the National Institute of Clinical Excellence [22]. Women with multiple pregnancies as well as patients with pre-pregnancy diabetes, cardiovascular disease or pre-eclampsia were excluded. The control group was defined as gestational age-matched women without abnormal OGTT.

Placentae were collected in the operating theatre and perfused within 10 min (*n* = 6 for both GDM and controls). Blood samples were collected in citrated plasma tubes (BD Vacutainer, Reading, UK). Peripheral blood samples were taken from the left antecubital fossa, while uterine vein blood samples were taken at caesarean section prior to delivery of the foetus. Samples were centrifuged at 1500 ×*g* for 15 min to separate cells from supernatant. The resulting platelet-poor plasma was immediately used for flow cytometry analysis.

Additional placental biopsies were taken through the maternal surface for protein expression analysis. Lysates were obtained with HEPES buffer and a protease inhibitor cocktail (Sigma Aldrich, Gillingham, UK), and subjected to a BCA protein assay (Sigma Aldrich, Gillingham, UK), before storage at −20°C until analysis.

### Immunohistochemistry

Placental tissues were fixed in 4% v/v formaldehyde, embedded in paraffin blocks, cut in 10 μm thick sections and placed on slides. Placental sections were deparaffinized in Histoclear (Sigma Aldrich, Gillingham, UK), and rehydrated in graded ethanol. For antigen retrieval, slides were heated in 10 mM sodium citrate at pH 6 for 10 min, and cooled down at room temperature (R/T). To limit background staining, endogenous peroxidase activity was blocked with 3% H_2_O_2_ in phosphate-buffered saline (PBS). Slides were rinsed with distilled water prior to blocking for non-specific antibody binding using 10% Fetal Calf Serum (FCS) (Sigma Aldrich, Gillingham, UK) in PBS-T (PBS with Tween-20) at R/T for 1 h. Sections were incubated overnight at 4°C with 1% FCS and 0.5 µg/ml anti-CD26 primary antibody (Abcam, Cambridge, UK) or non-immune mouse IgG1 (Biolegend, London, UK) in PBS-T. Sections were washed in PBS and incubated with anti-mouse IgG secondary antibody (Life Technologies, Paisley, UK) in a humidifying chamber at R/T for 1 h. Slides were washed with 0.01% PBS-T before being stained with DAB (Vector Laboratories, Peterborough, UK). All slides were washed in 0.01% PBS-T and distilled water before nuclei were counter-stained with Hematoxylin (Thermo Fisher, Paisley, UK) for 10 min. Slides were dehydrated in graded ethanol and Histoclear, mounted in Depex mounting medium (Sigma Aldrich, Gillingham, UK) and examined using a Leica DMIRE 2 microscope. Images were taken using Hamamatsu Orca digital camera and HCI software.

### Isolation and characterization of STB-EVs

STB-EVs were obtained from a placental dual lobe perfusion system as previously described [23]. Briefly, placentae were perfused for 3 h and the maternal side perfusate was collected and immediately centrifuged (Beckman Coulter Avanti J-20XP centrifuge and Beckman Coulter JS-5.3 swing out rotor) twice at 1500 ×*g* for 10 min at 4°C to remove erythrocytes and large cellular debris. Sequential centrifugations were performed to separate STB-EVs according to their size. The supernatant was collected and spun at 10,000 ×*g* (Beckman L80 ultracentrifuge and Sorvall TST28.39 swing out rotor) for 35 min at 4°C to pellet syncytiotrophoblast medium/large extracellular vesicles (MEDIUM/LARGE STB-EVs) (200 nm–1 µm in diameter). MEDIUM/LARGE STB-EVs were re-suspended in sterile PBS and the remaining supernatant was passed through a 0.2 µm Stericup filter (Millipore, Watford, UK) and spun at 150,000 ×*g* for 125 min at 4°C (Beckman L80 ultracentrifuge and Sorvall TST28.39 swing out rotor) to pellet smaller size syncytiotrophoblast extracellular vesicles (SMALL STB-EVs) (50–200 µm). SMALL STB-EVs were re-suspended in sterile PBS. Pellets containing MEDIUM/LARGE STB-EVs and SMALL STB-EVs (from the 10K and 150K centrifugations, respectively) were assessed for protein concentration using a BCA protein assay kit (Sigma Aldrich, Gillingham, UK). Particle size was characterized with Nanoparticle Tracking Analysis (NTA; Nanosight NS500, Malvern Instruments, Malvern, UK). MEDIUM/LARGE STB-EVs were also characterized using a BD LSRII flow cytometer (BD Biosciences, Wokingham, UK) as previously described [23]. STB-EVs were characterized by assessing the expression of STB-EVs-specific protein markers (specifically PLAP) to confirm syncytiotrophoblast origin and known markers of exosomes (e.g. syntenin, Alix and CD9).

### STB-EV size and particle characterization using NTA

STB-EVs diameter and concentration were measured using a NanoSight NS500 (Malvern Instruments, Malvern, UK) equipped with a sCMOS camera and the nanoparticle tracking analysis software version 2.3, Build 0033 (Malvern Instruments, Malvern, UK). The instrument was calibrated prior to our measurements, using silica 100 nm microspheres (Polysciences, Warrington, UK). Size distribution profiles and concentration of STB-EVs were measured using a protocol previously described [23].

### Immunoblotting

Placental lysates, MEDIUM/LARGE STB-EVs or SMALL STB-EVs (30 µg) were separated on Mini-Protean TGX NuPAGE gel cassettes (Bio-Rad, Oxford, UK). Proteins were transferred onto a polyvinylidene difluoride membrane (Bio-Rad Laboratories, Watford, UK) in a Novex Semi-Dry Blotter (Life Technologies, Paisley, UK). Membranes were blocked for 1 h at R/T in 5% (w/v) Blotto (Alpha Diagnostic, Eastleigh, UK) in TBS-T (Tris-buffered saline solution with 0.1% Tween-20) and incubated with primary antibodies: anti-DPPIV/CD26 antibody (1 µg/ml) (R&D System, Abingdon, UK) or anti-PLAP (1 µg/ml) (NDOG-2, In-house) overnight, at 4°C. Membranes were washed three times in TBS-T and incubated with horseradish peroxidase (HRP)-conjugated secondary antibodies (Life Technologies, Paisley, UK) in Blotto/0.1% TBS-T for 1 h at R/T. Membranes were washed three times in TBS-T and antigen–antibody complexes were visualized by chemiluminescence using an ECL kit (ECL Western Blotting substrate, Thermo Scientific, Paisley, UK).

### Magnetic bead depletion

Dynabeads M-280 Sheep Anti-Mouse IgG (Life Technologies, Paisley, UK) conjugated to anti-DPPIV (anti-CD26) antibody (Biolegend, London, UK) or anti-PLAP antibody were prepared according to the manufacturer’s instructions. Dynabeads coated with anti-IgG1 antibody (Biolegend, London, USA) or anti-IgG2a antibody (Dako, Ely, UK) were used as controls. Briefly, Dynabeads (50 µl) were washed in Ca^2+^ and Mg^2+^-free PBS supplemented with 0.1% BSA and 2 mM EDTA at pH 7.4, pelleted with a magnet, and re-suspended in the same buffer. Beads were incubated with 6 µg of either anti-PLAP, anti-DPPIV, anti-IgG1 or anti-IgG2a antibody on a rotating plate overnight, at 4°C. The beads were aggregated using a magnet and supernatants were discarded. MEDIUM/LARGE STB-EVs or SMALL STB-EVs (25 µg for both) were incubated with 10 µl of anti-human Fc receptor blocking reagent for 10 min at 4°C prior to incubation with the beads overnight at 4°C for depletion. Bound and unbound STB-EVs were separated using a magnetic separator (Dynal, Oslo, Norway). Pellets containing bound antigen-positive STB-EVs were immunoblotted. Supernatants (antigen-negative STB-EVs) were analysed by NTA to calculate the percentage of STB-EVs bound to the beads as follows:

$$\;\;\;\;\;\;\;\;\;\;\;\;\;\;\;\;\;\;\;\;\;\;\;\;\;\;\;\;\;\;\;\;\;\;\;\;\;\;\;\;\;\;\;\;\;\;\;\;\;\;\;\;\;\;\;\;\;\;\;\;\;\;\;\;\;\;\;\;\;\;\;\;\;\;\;\;\;\;\;\;\;\;\;\;\;\;\;\;\;\;\%DPPIV\;or\;PLAP=\frac{\left[Total\right]-\left[DPPIV\;or\;PLAP\;negative\right]}{\left[Total\right]}*100$$

### Flow cytometry analysis

#### MEDIUM/LARGE STB-EVs analyses

MEDIUM/LARGE STB-EVs from 10K centrifugation spin were characterized using a BD LSRII flow cytometer (BD Biosciences, UK). TruCount tubes, containing a known number of fluorescent beads suspended in 500 µl of filtered PBS, were used to establish the flow rate. The background event rate was set at <1000 events/min. MEDIUM/LARGE STB-EV samples were diluted in filtered PBS to optimize an event rate of ∼300 events/s in a final volume of 300 µl. Prior to staining, samples were incubated with 10 µl of Fc receptor blocker (Miltenyi, Woking, UK) for 10 min, at 4°C. Samples were then labelled with anti-PLAP-PE, anti-DPPIV-Alexa Fluor 647 and Bio-maleimide-FITC (used as an MEDIUM/LARGE STB-EV membrane marker (BODIPY FL N-(2-aminoethyl)-maleimide); Thermo Fisher, Paisley, UK) for 15 min at R/T. Isotype controls were matched to their respective antibodies according to the concentration, fluorochrome type and heavy chain. Prior to data acquisition, final volumes were adjusted to 300 µl with PBS. A total of 100,000 events were collected for each sample. EV gates were set at <1 µm using fluorescent beads. The negative gates for staining were determined using isotype control tests and set at 1%; both data analyses and figure generation were carried out using FlowJo version 10.1 (Tree Star Inc, Ashland, OR, USA).

#### Plasma sample analyses

Plasma PLAP- and DPPIV-positive EVs were identified using a four-colour flow cytometry protocol previously described [24], but with additional modifications to exclude contamination from other EV sources, by defining a “dump-channel” [25]. The “dump channel” excluded the principal contaminants such as platelet EVs (using anti-CD-41a-PE-Cy7), erythrocyte EVs (using anti-CD235a-PE-Cy7) and EVs derived from all other sources except STB or erythrocytes (using anti-HLA Class I-PE-Cy7 and anti-HLA Class II-PE-Cy7). Hundred microlitres of platelet-poor plasma was labelled with these antibodies, together with anti-PLAP-PE and anti-DPPIV-Alex-Fluor 647 for 15 min, at 4°C. Labelled plasma samples were subsequently filtered using Ultrafree-MC/Durapore-PVDF (Millipore, Watford, UK) centrifugal filters (2 min, 800 ×*g*). MEDIUM/LARGE STB-EVs were recovered from the top of the filter unit in 100 µl of filtered PBS, and stained with Bio-maleimide-FITC, which stains biological membranes. Volumes were then adjusted to 500 µl with filtered PBS, and each sample was analysed for 10 min by flow cytometry. Filtrate (negative for Bio-maleimide) was used to define control gates for Bio-maleimide and the “Dump Channel” in order to separate the population positive for Bio-maleimide and negative for contaminates. Events that fell into this gate (i.e. biological membranes but not contaminants) were further analysed for PLAP and DPPIV signal. To determine control gates for anti-DPPIV-Alexa Fluor 647 and anti-PLAP-PE staining, samples were treated with detergent Nonidet P-40 (Sigma Aldrich, Gillingham, UK) (to destroy the vesicle lipid membrane) and the gates were set at 1% cut-off. Both data analyses and figure generation were carried out using a FlowJo version 10.1.

### DPPIV enzyme activity assay

DPPIV enzyme activity of the STB-EVs was determined using a DPPIV-Glo Protease Assay (Promega, Southampton, UK). Briefly, the luminescent DPPIV-Glo Reagent was incubated at R/T for 30 min with either clinical samples (STB-EVs isolated from perfused placentae from both GDM and normal pregnancy), Tris-BSA (blanks) or a purified DPPIV enzyme in Tris-BSA as standard (Recombinant human CD26 protein, Abcam, Cambridge, UK). Luminescence was measured using a FLUOstar Omega apparatus (BMG Labtech, Aylesbury, UK).

For the DPPIV enzymatic inhibition studies, MEDIUM/LARGE STB-EVs and SMALL STB-EVs were pre-treated with vildagliptin (DPPIV-specific inhibitor) (Sigma Aldrich, Gillingham, UK) (0–100 nM) for 1 h, and DPPIV residual enzymatic activity was determined.

STB-EV-bound DPPIV ability to break GLP-1 was measured using a GLP-1 active assay according to manufacture instructions (Cisbio, Codolet, France). MEDIUM/LARGE STB-EV and SMALL STB-EV pools of three GDM patients in series dilution were mixed with 1400 pg/ml of GLP-1, respectively, and incubated with anti-Active GLP-1-Tb3+Cryptate Antibody and with anti-Active GLP-1-d2 antibody overnight at RT. Recombinant DPPIV (Recombinant human CD26 protein, Abcam, Cambridge, UK) was used as positive control, and PBS was used as negative control. HTRF measurement was performed using a CLARIOstar apparatus (BMG Labtech, Aylesbury, UK).

### Statistics

Results were statistically analysed using Prism 3.0 (GraphPad Software Inc, San Diego, CA, USA). Comparisons of means between two groups were tested using the independent two-sample Student’s *t*-test, with statistical significance assigned when *p < 0.05. Paired samples were compared with the Wilcoxon matched-pairs signed rank test. Unpaired samples were analysed using the Mann–Whitney test.

## Results

### DPPIV is present in normal STB-EVs

**Figure 1.**
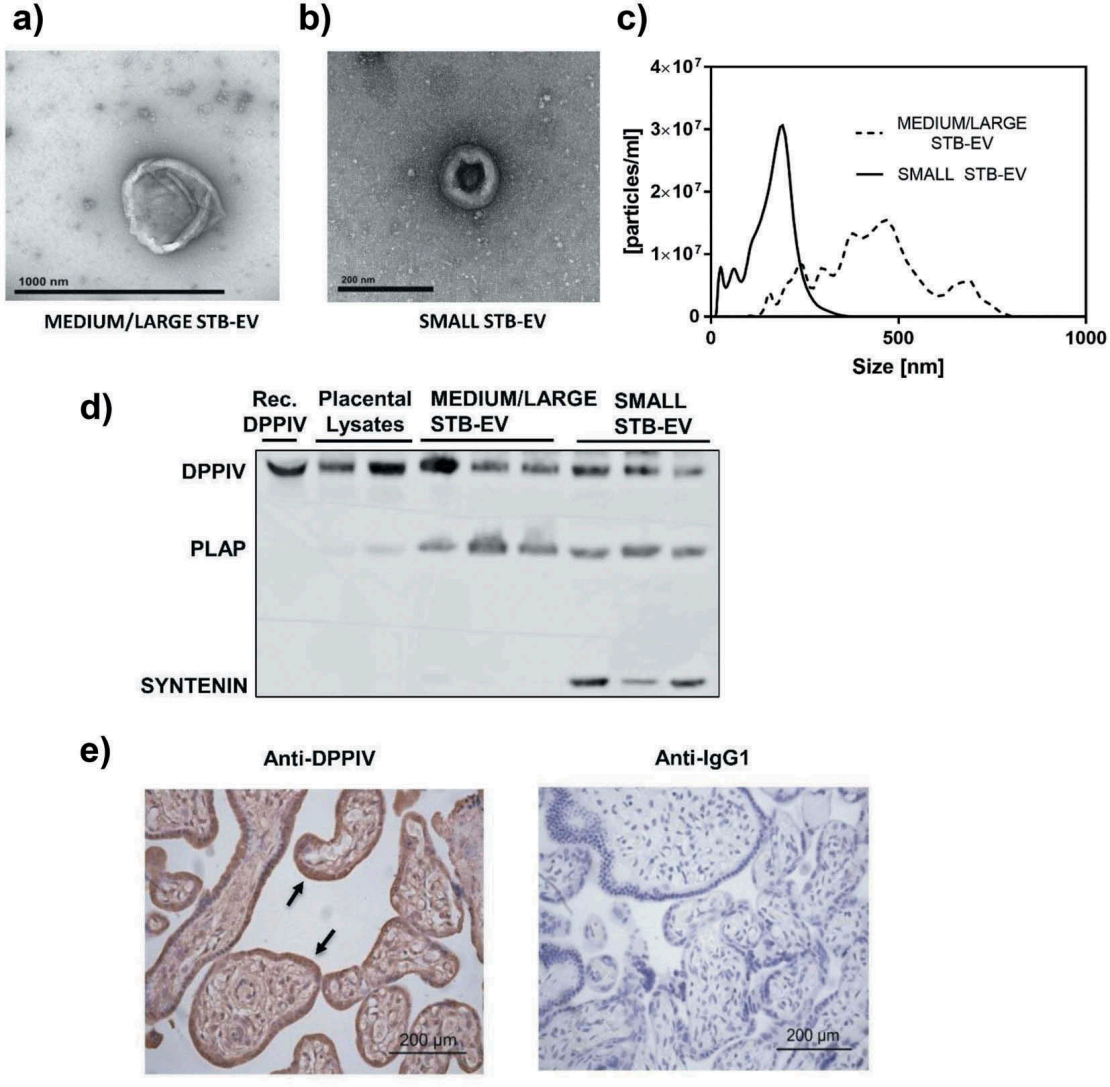
DPPIV is expressed in normal placentae, MEDIUM/LARGE STB-EVs and SMALL STB-EVs. Transmission electronic microscopy of MEDIUM/LARGE STB-EVs (scale bar represents 1000 nm) (a) and SMALL STB-EVs (b) (scale bars represent 200 nm) isolated from normal placentae. (c) Representative Nanoparticle Tracking Analyses (NTA) size vs. distribution profiles of MEDIUM/LARGE STB-EVs and SMALL STB-EVs from normal pregnancies. (d) Representative Western blot (*n* = 3) of DPPIV protein expression in whole placental lysates, MEDIUM/LARGE STB-EVs and SMALL STB-EVs isolated from control term placentae. Recombinant human DPPIV protein (Rec. DPPIV) was used as positive control. PLAP expression confirmed syncytiotrophoblast origin; expression of exosomal marker syntenin was enriched in SMALL STB-EVs. Blots are representative of *n* = 3 separate experiments. (e) Control placental sections were subjected to immunohistochemical analysis for DPPIV expression (left panel). DPPIV positivity is seen at the syncytiotrophoblast (arrow). As negative controls, placental sections were incubated with anti-IgG1 antibody (right panel). Images are representative of *n* = 3 separate experiments. Scale bar sets at 200 µm.

STB-EVs were obtained after perfusion of placentae of control/normal pregnancies. Transmission electron microscopy analyses revealed particles with physical characteristics consistent with medium/large EV (MEDIUM/LARGE STB-EVs) and small EV (SMALL STB-EVs) in pellets collected after 10K and 150K centrifugations of the perfusates, respectively (Figure 1(a,b)). NTAs confirmed the heterogeneous size of the MEDIUM/LARGE STB-EV population (approximately 0.2–1 µm) whilst the SMALL STB-EVs were more homogenous particles with a smaller size range (approximately 50–200 nm) (Figure 1(c)).

The syncytiotrophoblast-specific marker PLAP was expressed in both MEDIUM/LARGE STB-EVs and SMALL STB-EVs, confirming the placental origin of these two particle populations (Figure 1(d)); and the expression of exosome-specific marker syntenin was enriched in the SMALL STB-EVs fraction (Figure 1(d)), with marginal expression in MEDIUM/LARGE STB-EVs (Fig. S1). Expression of known exosomal markers Alix and CD9 were also found enriched in the SMALL STB-EVs fraction (Fig. S1).

Our previous mass spectrometric analyses suggested the presence of DPPIV, a glycoprotein that rapidly cleaves and inhibits the incretin hormone GLP-1, in MEDIUM/LARGE STB-EVs of normal pregnancies [26]. We now confirmed DPPIV protein expression in both MEDIUM/LARGE STB-EVs and SMALL STB-EVs of normal pregnancies (Figure 1(d) upper band) using specific immunoblotting. Consistent with the literature, we detected DPPIV in the whole placental tissue, with a signal localized in the syncytiotrophoblast layer (Figure 1(e)), demonstrating that DPPIV was present at the site of STB-EVs production [26,27].

### DPPIV is co-expressed with PLAP on MEDIUM/LARGE STB-EVs and SMALL STB-EVs

**Figure 2.**
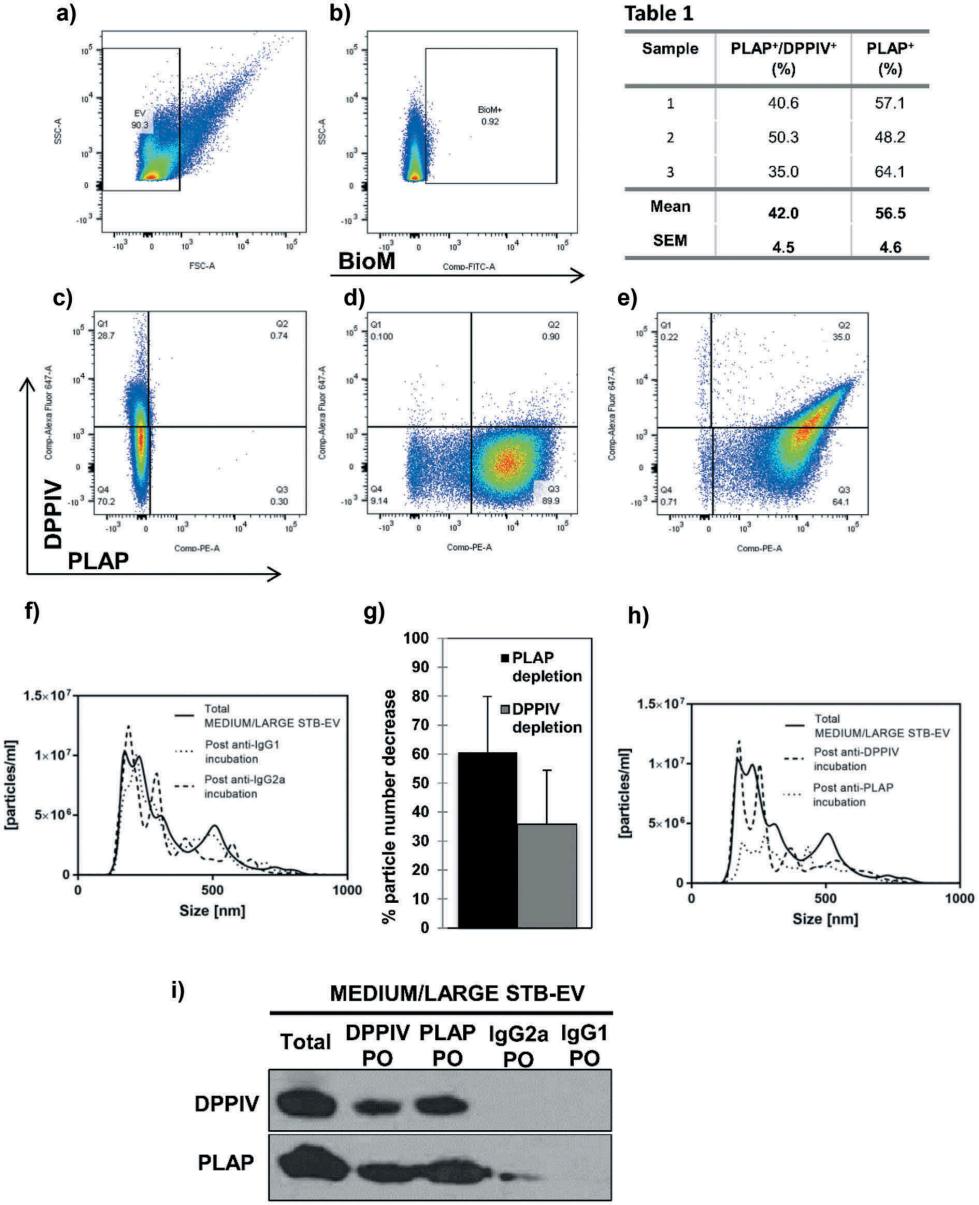
DPPIV and PLAP co-expression in MEDIUM/LARGE STB-EVs. Representative image of flow cytometry analysis of MEDIUM/LARGE STB-EV (*n* = 3) derived from dual placental lobe perfusion showing (a) forward scatter (FSC) versus side scatter (SSC) dot plot of MEDIUM/LARGE STB-EVs. Fluorescent microspheres of 1 μm (Polysciences Inc., Warrington, PA, USA) were used to establish a gate <1 μm. Events that fell into this gate were subject to further analysis. (b) Bio-maleimide (BioM) fluorochrome minus one (FMO) was used to draw 1% cut-off gate for BioM-positive events. (c) PLAP FMO was used to draw 1% cut-off gate for PLAP-positive events. (d) DPPIV FMO was used to draw 1% cut-off gate for DPPIV-positive events. (e) The co-expression of PLAP-PE and DPPIV-Alexa Fluor 647 on MEDIUM/LARGE STB-EVs (Quadrant 2). (f) Representative Nanoparticle Tracking Analysis (NTA) size distribution profile of total MEDIUM/LARGE STB-EVs, MEDIUM/LARGE STB-EVs population depleted of MEDIUM/LARGE STB-EVs expressing DPPIV and MEDIUM/LARGE STB-EVs expressing PLAP. (g) Percentage of particle number after PLAP or DPPIV depletion. (h) Representative NTA size distribution profile of total MEDIUM/LARGE STB-EVs, MEDIUM/LARGE STB-EVs population depleted of MEDIUM/LARGE STB-EVs expressing IgG2a and MEDIUM/LARGE STB-EVs population depleted of MEDIUM/LARGE STB-EVs expressing IgG1. Graphs are representative of *n* = 3 experiments. (i) DPPIV and PLAP protein expression in total MEDIUM/LARGE STB-EVs (Total), in MEDIUM/LARGE STB-EVs population pulled out with anti-DPPIV magnetic beads (DPPIV PO), in MEDIUM/LARGE STB-EVs population pulled out with anti-PLAP magnetic beads (PLAP PO), in MEDIUM/LARGE STB-EVs population pulled out with anti-IgG2a magnetic beads (IgG2a PO) and in MEDIUM/LARGE STB-EVs population pulled out with anti-IgG1 magnetic beads (IgG1 PO). Blot is representative of *n* = 3 experiments.

Flow cytometry analysis was performed on MEDIUM/LARGE STB-EVs isolated with 10K centrifugation of maternal perfusate (Representative data shown in Figure 2(a–e)). We first established an EV positive gate (Figure 2(a)) using fluorescent microspheres of 1 μm. Events that fell into this gate were subjected to further analysis. Bio-maleimide positive gate was defined by fluorescence minus one (FMO) (Figure 2(b)). PLAP FMO and isotype control and DPPIV FMO and isotype control were used to draw 1% cut-off gates for PLAP-positive events (Figure 2(c)) and DPPIV-positive events (Figure 2(d)), respectively. In Figure 2(e), the significant co-expression of PLAP and DPPIV is demonstrated in quadrant 2. We noted PLAP-positive and DPPIV-negative events in quadrant 3 as well as a very small (0.22%) population of DPPIV-positive PLAP-negative events in quadrant 1. Three separate analyses are shown in Table 1. More than half of MEDIUM/LARGE STB-EVs (56.5 ± 4.6%) were solely PLAP positive. Importantly, 42 ± 4.5% of the MEDIUM/LARGE STB-EVs were both PLAP and DPPIV positive, suggesting that a large proportion of MEDIUM/LARGE STB-EVs express both PLAP and DPPIV in control pregnancies.

**Table 1.** DPPIV is co-expressed with PLAP on MEDIUM/LARGE STB-EVs.

Sample	PLAP^+^/DPPIV^+^ (%)	PLAP^+^/DPPIV^−^ (%)
1	40.6	57.1
2	50.3	48.2
3	35.0	64.1
**Mean**	**42.0**	**56.5**
**SEM**	**4.5**	**4.6**

Percentage of PLAP/DPPIV positivity and PLAP positivity in MEDIUM/LARGE STB-EVs isolated from human placentae by dual-lobe placental perfusion using flow cytometry in three independent biological samples. A total of 100,000 events recorded with data presented as mean ± SEM.

In addition, total MEDIUM/LARGE STB-EVs were incubated with either anti-DPPIV- or anti-PLAP-coated magnetic beads and pulled out using a magnet. Particle numbers in the DPPIV- or PLAP-depleted supernatants were determined by NTA. Figure 2(f,g) revealed that PLAP depletion decreased particle numbers by 60.61 ± 19.31%; and DPPIV depletion decreased particle numbers by 35.74 ± 18.65%, essentially confirming the fluorescence activated cell sorting (FACS) analyses. Control experiments using IgG1- or IgG2a-coupled magnetic beads did not alter MEDIUM/LARGE STB-EVs numbers (Figure 2(h)).

DPPIV or PLAP pull-out fractions were analysed by immunoblotting to assess co-expression of DPPIV and PLAP protein (Figure 2(i)). PLAP was found in the DPPIV pull-out fractions and conversely, DPPIV was found in PLAP pull-out fractions, confirming co-expression on both MEDIUM/LARGE STB-EVs population. When MEDIUM/LARGE STB-EVs were incubated with magnetic beads coated with PLAP, pull-out showed a greater signal for DPPIV compared with the pull-out from magnetic beads coated with DPPIV suggesting that some DPPIV is intravesicular.

**Figure 3.**
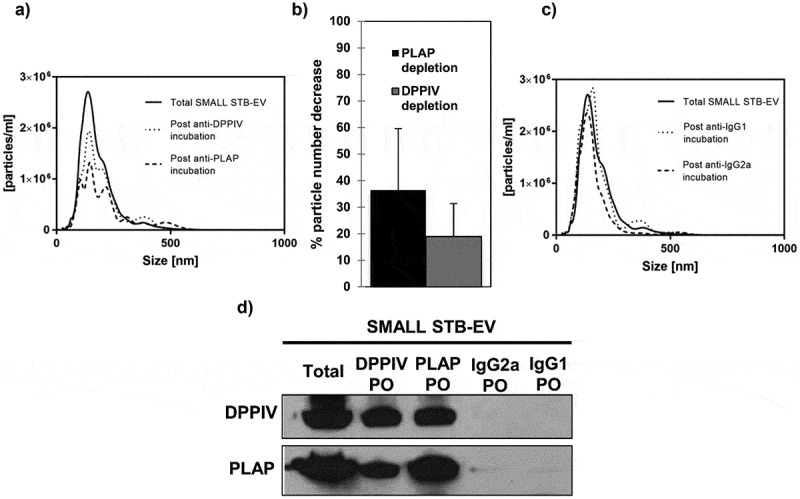
DPPIV and PLAP co-expression in SMALL STB-EVs. (a) Representative Nanoparticle Tracking Analysis (NTA) size distribution profile of total SMALL STB-EVs, SMALL STB-EVs population depleted of SMALL STB-EVs expressing DPPIV and SMALL STB-EVs expressing PLAP. (b) Percentage of particle number after PLAP or DPPIV depletion. (c) NTA size distribution profile of total SMALL STB-EVs, SMALL STB-EVs population depleted of SMALL STB-EVs expressing IgG2a and SMALL STB-EVs population depleted of SMALL STB-EVs expressing IgG1. Graphs are representative of *n* = 3 experiments. (d) DPPIV and PLAP protein expression in total SMALL STB-EVs (Total), in SMALL STB-EVs population pulled out with anti-DPPIV magnetic beads (DPPIV PO), in SMALL STB-EVs population pulled out with anti-PLAP (PLAP PO), in SMALL STB-EVs population pulled out with anti-IgG2a magnetic beads (IgG2a PO) and in SMALL STB-EVs population pulled out with anti-IgG1 magnetic beads (IgG1 PO). Blot is representative of *n* = 3 experiments.

Lastly, essentially similar data were obtained for SMALL STB-EVs. SMALL STB-EVs could not be analysed by flow cytometry, as their size is below the limit of detection. Therefore, particle numbers in the DPPIV- or PLAP-depleted SMALL STB-EVs supernatants were determined by NTA. PLAP depletion decreased particle numbers by 36.45 ± 23.01% and DPPIV depletion decreased particle numbers by 18.94 ± 12.42% (Figure 3(a,b)). Control experiments did not alter SMALL STB-EV numbers (Figure 3(c)). Additionally, we confirmed that DPPIV and PLAP proteins were also found in the same population of SMALL STB-EVs (Figure 3(d)).

### DPPIV present in STB-EVs displays enzymatic activity

**Figure 4.**
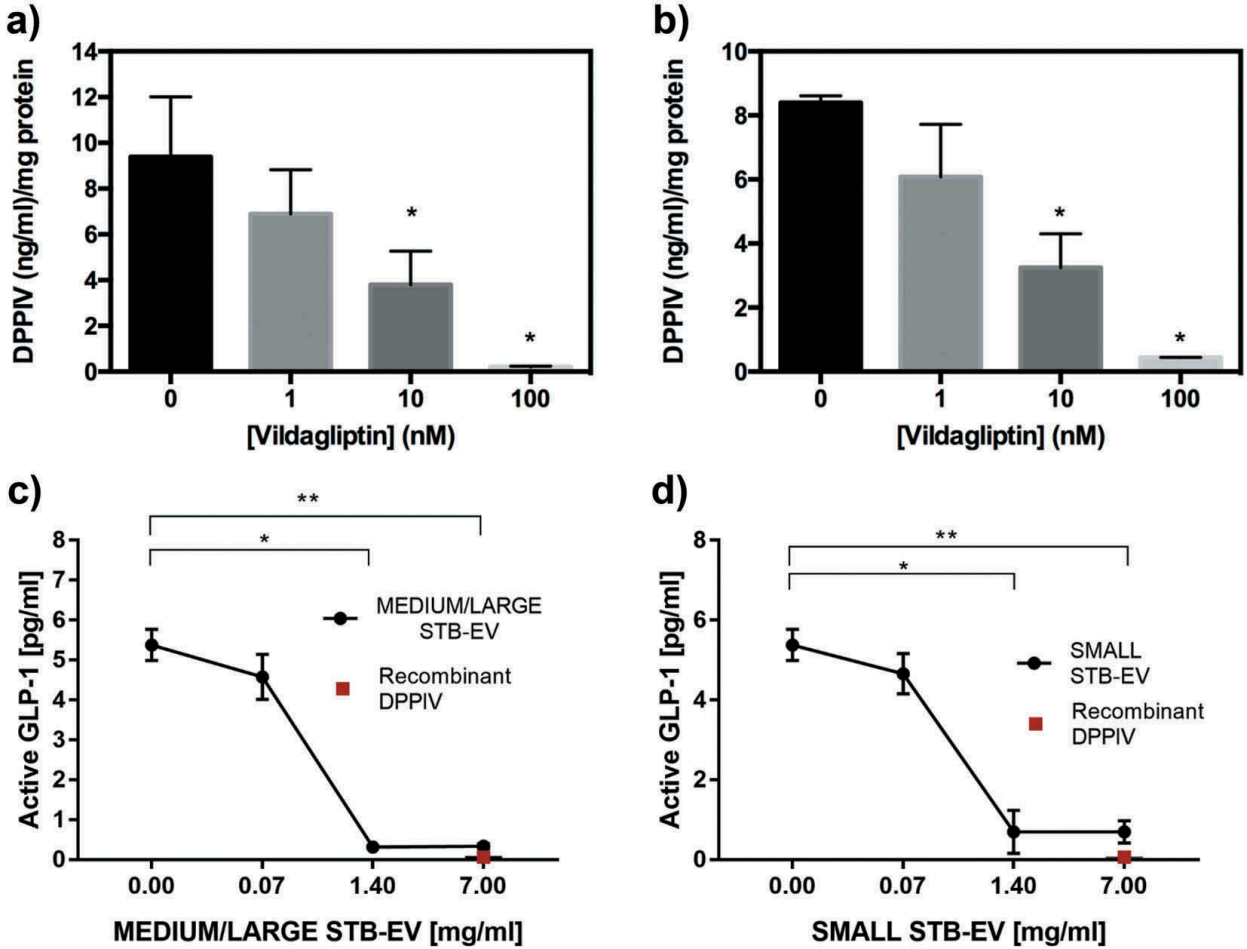
DPPIV in MEDIUM/LARGE STB-EVs and SMALL STB-EVs is enzymatically functional. (a) MEDIUM/LARGE STB-EVs and (b) SMALL STB-EVs from control women were incubated with 0, 1, 10 or 100 nM vildagliptin (inhibitor of DPPIV) and DPPIV activity was measured. Percentage of residual DPPIV activity was calculated in (a) MEDIUM/LARGE STB-EVs and (b) SMALL STB-EVs. Graphs are representative of *n* = 3 experiments performed independently in duplicates (*p < 0.05). (c) MEDIUM/LARGE STB-EVs and(d) SMALL STB-EVs pools from three GDM women were diluted to 7, 1.4 or 0.07 mg/ml and incubated with 1400 pg/ml of standard GLP-1. Graphs show levels of active GLP-1 measured after the incubation with MEDIUM/LARGE STB-EVs or SMALL STB-EVs. Recombinant DPPIV was used as positive control. Graphs are representative of *n* = 3 experiments performed independently in duplicates (*p < 0.05).

Surface-bound DPPIV activity was next determined in MEDIUM/LARGE STB-EVs and SMALL STB-EVs of control pregnancies. Basal DPPIV activity was similar in MEDIUM/LARGE STB-EVs and SMALL STB-EVs (Figure 4(a,b)). Importantly, incubation with the DPPIV-specific inhibitor vildagliptin inhibited DPPIV activity in both MEDIUM/LARGE STB-EVs and SMALL STB-EVs in a dose-dependent manner, demonstrating that placental EVs expressed functional DPPIV.

**Figure 5.**
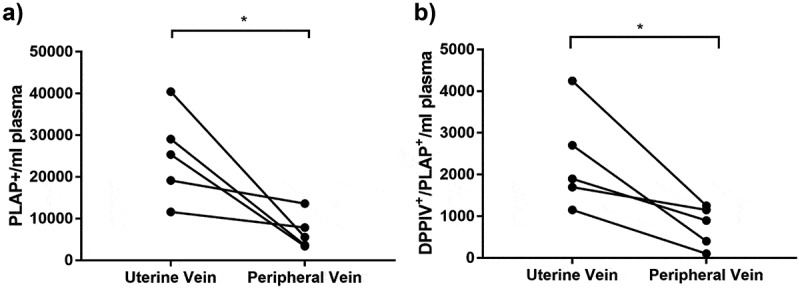
DPPIV expressing STB-EVs are present in the circulation and are placentally derived. Paired plasma samples were collected from the uterine and the peripheral vein of pregnant women. (a) Individual PLAP-positive event numbers from matched peripheral and uterine vein plasma samples (*n* = 5 women; *p < 0.05). (b) Individual PLAP/DPPIV double-positive event numbers from matched peripheral and uterine vein plasma samples (*n* = 5 women; *p < 0.05).

Furthermore, we demonstrated that functional DPPIV present on STB-EVs and SMALL STB-EVs can degrade active GLP-1. Figure 5 shows that increasing concentrations of MEDIUM/LARGE STB-EVs (Figure 4(c)) and SMALL STB-EVs (Figure 4(d)) resulted in a dose-dependent decrease of active GLP-1.

### DPPIV expressing STB-EVs in the maternal circulation

The concentration of DPPIV-positive STB-EVs in uterine vein (which drains the utero-placental unit) was compared to peripheral venous blood in the same women (*n* = 5) in order to confirm its release from the placenta. Briefly, during routine caesarean section, blood samples were collected from the uterine vein (after reflection of the bladder and before uterine incision and prior to delivery of the foetus) in order to obtain blood as soon as it left the utero-placental unit. Concomitantly blood was also taken from the left ante-cubital fossa (peripheral vein) from the same patient. Both samples were tested for presence of placental EVs expressing both DPPIV and PLAP by flow cytometry. As expected, concentrations of single PLAP-positive STB-EVs were higher in uterine (25,210 ± 4842.20 events/ml) than peripheral (6810 ± 1677.94 events/ml) venous plasma of these pregnant women (Figure 5(a)).

Importantly, a similar gradient of PLAP and DPPIV double-positivity (Figure 5(b)) was observed between uterine (2340 ± 538.38 events/ml) and peripheral (760 ± 197.69 events/ml) blood confirming placental origin of these double-positive EVs. This gradient was present in all samples. Representative flow cytometry dot plot is available in the supplement (Fig. S2).

### DPPIV activity is increased in STB-EVs of GDM pregnancies

**Figure 6.**
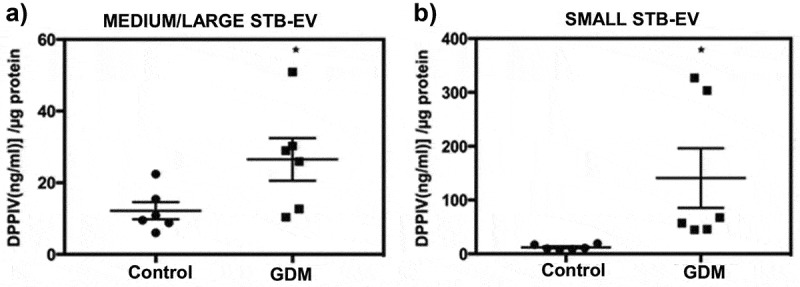
DPPIV activity is increased in STB-EVs of GDM pregnancies. (a) DPPIV concentration in MEDIUM/LARGE STB-EVs from GDM versus control isolated from placental perfusions (*n* = 6 for both groups; *p < 0.05). (b) DPPIV concentration in SMALL STB-EVs from GDM versus control isolated from placental perfusions (*n* = 6 for both groups; *p < 0.05).

We compared DPPIV activity in placental EVs isolated from normal and GDM perfused placentae (*n *= 6 for both). The clinical characteristics of the patients from whom STB-EVs were analysed are described in Supplementary Table S1. MEDIUM/LARGE STB-EVs from GDM placentae showed significant twofold increase in DPPIV activity compared to MEDIUM/LARGE STB-EVs from controls (Figure 6(a)). This increased DPPIV activity was also seen in SMALL STB-EVs from GDM placentae and was fivefold higher than that found in controls (Figure 6(b)).

### STB-EV-bound DPPIV expression is increased in GDM peripheral plasma

**Figure 7.**
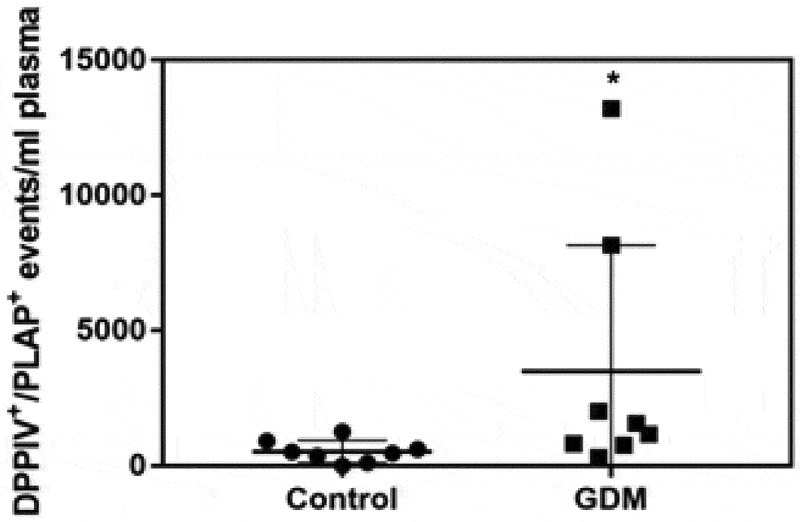
Placenta-derived DPPIV expression is increased in GDM peripheral plasma. Peripheral gestation-matched plasma samples were collected from GDM (*n* = 8) and Control (*n* = 8) pregnant women. DPPIV and PLAP double-positive events in 1 ml of plasma were identified by flow cytometry. *P < 0.0157.

Lastly, we investigated whether there were differences in MEDIUM/LARGE STB-EVs expressing DPPIV in the plasma of women with GDM. Double-positive PLAP and DPPIV STB-EV levels in peripheral blood were 8.58-fold increased in women with GDM, compared to control women (Figure 7). Patient characteristics are presented in Supplementary Table S1.

## Discussion

In this study, we investigated (1) the presence and bioactivity of the well-established regulator of insulin secretion DPPIV in STB-EVs during pregnancy and (2) whether DPPIV concentration and/or activity was changed in pregnancies complicated with GDM.

We found that the normal human placenta releases STB-EVs that carry biologically active DPPIV. This DPPIV was shown to have intrinsic enzymatic activity and was also shown to degrade active GLP-1. We further showed that GDM pregnancies are associated with a greater than eightfold increase in DPPIV-positive STB-EVs concentration in maternal plasma, compared to normal pregnancies. Increased DPPIV biological activity was also demonstrated in both MEDIUM/LARGE STB-EVs and SMALL STB-EV from GDM placentae, compared to controls.

EVs are now understood to play important roles in mediating intercellular communication as well as regulating biological signalling in target cells [19,28]. The concentrations of STB-EVs released by the placenta increase during normal pregnancy [13], which correlates with the physiological establishment of insulin resistance in normal pregnancies. In addition, the number of SMALL STB-EVs present in maternal plasma is reported to be significantly increased in pregnancies complicated with GDM [18]. Consistent with the hyperglycaemia observed in GDM, glucose concentrations have been shown to increase exosome release from trophoblast cells *in vitro* [29]. Thus, for the reasons above we hypothesized that the composition of the STB-EVs itself might be changed during GDM. We focused on possible changes in molecules known to impact insulin sensitivity or secretion, both of which are altered in pregnancies complicated with GDM.

In 2000, our group performed a mass spectrometric analysis of mechanically isolated MEDIUM/LARGE STB-EVs in attempt to identify complexes that might inhibit proliferation of endothelial cells [26]. One of the molecules identified was DPPIV. We now demonstrate that not only is DPPIV expressed in STB-EVs, with increased concentration in GDM pregnancies, but also that DPPIV is biologically active and can be inhibited using a specific inhibitor. This differs from Liu et al. [30] who reported no difference in DPPIV concentration between normal and GDM pregnancies. However, whilst Liu et al. measured the enzymatic activity of soluble DPPIV, we directly measured DPPIV activity bound to STB-EVs, which might explain the divergence. Another possible explanation is that they used serum for analysis in their study, which is not the most suitable medium for EV analysis [31]. Jayabalan et al. [32] analysed plasma exosomes from normal and GDM women at diagnosis and showed that the overall DPPIV exosome levels were downregulated, but this analysis did not target placental EVs specifically nor did it analyse medium/large EVs.

DPPIV plays a major role in glucose metabolism by regulating the secretion of insulin. In particular, DPPIV rapidly degrades GLP-1 (a member of the incretin family), consequently decreasing glucose-dependent insulin secretion [21]. Decreased insulin secretion is also noted in GDM pregnancies [10]. Reduced GLP-1 concentration after meal uptake was described by Bonde et al. in both normal and GDM pregnancies, but with significantly higher decreases seen in GDM pregnancies [33]. Supporting this, we have shown that STB-EVs can decrease active GLP-1. We also showed that DPPIV activity, although present in both normal and GDM pregnancies, was increased in GDM pregnancies. This might provide a possible explanation for the aforementioned decline of GLP-1 observed in GDM. Additionally, the reported greater numbers of SMALL STB-EVs in GDM could also contribute to increased breakdown of GLP-1 in GDM pregnancies. Interestingly, GLP-1 function rapidly returns to normal in both GDM and normal pregnancies after delivery [33], showing that the effect is remarkably transient. Our finding is consistent with the hypothesis that STB-EVs could reduce incretin bioavailability during gestation by delivering active membrane-bound DPPIV, which decreases postpartum as the release of STB-EVs declines following placental delivery [34].

Whilst increased DPPIV in STB-EVs of GDM pregnancies might impact insulin sensitivity via the regulation of GLP-1, other mechanisms could also be considered. For example, a recent randomized and double-blind trial demonstrated that the DPPIV inhibitor sitagliptin attenuated insulin resistance and improved overall glycaemic control in women with GDM by a purported reduction in RBP4 (Retinol Binding Protein 4) levels [35]. However, DPPIV levels were not determined in this study.

Finally, DPPIV is a multifunctional protein implicated in the regulation of chemokine and cytokine activities. GDM is also associated with an imbalance between circulating pro- and anti-inflammatory cytokines. Isolated SMALL STB-EV from GDM pregnancies increases cytokine release from endothelial cells to a greater extent than those of control pregnancies [36]. Therefore, it is possible that increased DPPIV activity in GDM pregnancies might also affect the release or the activity of circulating of pro- or anti-inflammatory factors, which have been implicated in the dysregulation of insulin signalling and the establishment of insulin resistance. Further work to dissect this is required.

Limitations of the study include the following: the number of patients we have analysed is small, but the isolation and characterization of these STB-EVs is time intensive and complex. Our flow cytometry analysis (due to the instrument’s limit of detection) focuses solely on medium/large EVs. We accept that not all medium/large EVs are analysed in this way – however, this is the same for all samples. The data presented here are obtained from term pregnancies and it would be important to investigate the levels of STB-EVs DPPIV earlier in pregnancy, since the presence of STB-EV DPPIV might allow earlier diagnosis if the elevation seen in GDM occurs earlier in pregnancy. A larger study might allow us to understand the contribution of maternal characteristics (e.g. obesity, ethnicity) to the circulating DPPIV levels and thus allow stratification of severity.

We have demonstrated that STB-EVs express DPPIV, which is not only active but also present in higher quantities in the maternal circulation. This raises the possibility of their use as a potential diagnostic tool. A larger study would be required to further validate this approach as a potential means of early GDM screening/diagnosis.
