# Supplementary material for: Placental extracellular vesicles express active dipeptidyl peptidase IV; levels are increased in gestational diabetes mellitus
Source: J Extracell Vesicles. 2019 May 23;8(1):1617000. doi: 10.1080/20013078.2019.1617000 (PMC6534242; doi:10.1080/20013078.2019.1617000)
Supplement: Supplemental Material [file ZJEV_A_1617000_SM1600.zip › Supplemental data.docx]

|  | Normal Pregnancy (n=14) | | GDM Pregnancy (n=14) | |
| --- | --- | --- | --- | --- |
|  | **Plasma**  **STB-EV (n=8)** | **Placental Perfusion STB-EV (n=6)** | **Plasma**  **STB-EV (n=8)** | **Placental Perfusion STB-EV (n=6)** |
| Age, year | 37.8 ± 2.4  (24 - 45) | 35.3 ± 2.4  (28 - 43) | 37.4 ± 2.3  (33 - 42) | 29.8 ± 3.2  (22 - 39) |
| Gestation age, weeks | 39.3 ± 2.9  (38.3 – 41) | 39 ± 0.8  (38.6 – 39.4) | 39.5 ± 1.5  (39.1 – 40.6) | 38.7 ± 2.4  (37.3 – 39.4) |
| Body mass index, kg/m2 | 26.7 ± 1.5*  (22 – 34.5) | 30.2 ± 3.2*  (25 – 43) | 36.4 ± 1.7  (31 - 44) | 34.3 ± 3.0  (26 - 45) |
| Newborn weight, g | 3656.3 ± 219.3  (2890 - 4550) | 3630.8 ± 172.7  (2890 - 4550) | 3864.3 ± 231.4  (2790 – 4500) | 3994.2 ± 261.2  (2790 – 4500) |
| Max. systolic pressure, mmHg | 129.6 ± 2.8  (119 - 140) | 133.8 ± 3.2  (120 - 140) | 135 ± 4.8  (120 - 151) | 139.8 ± 6.4  (127 - 170) |
| Max. diastolic pressure, mmHg | 77.4 ± 3.3  (68 - 95) | 86.5 ± 4.5  (72 - 100) | 84.4 ± 3.8  (74 - 100) | 85.3 ± 1.7  (79 - 90) |
| GDM  Treatment | - | - | Diet: 2  Metf: 4  Ins + Metf: 2 | Diet: 2  Metf: 2  Ins: 1  Ins + Metf: 1 |

**Sup****plementary Table 1: Patient Characteristics**

Data is presented as mean ± SEM. Range is also included. Significant difference shown as * if p<0.05.

Metf, metformin; Ins, insulin.

**Supplementary Figure 1S. Characterisation of normal pregnancy derived syncytiotrophoblast medium/large extracellular vesicles (MEDIUM/LARGE STB-EVs) and small extracellular vesicles (SMALL STB-EVs) by western blotting.**

**(A)** Exosomal markers Alix, Syntenin and CD9 expression in whole placental lysates, MEDIUM/LARGE STB-EVs and SMALL STB-EVs isolated from control term placentae. PLAP expression confirmed syncytiotrophoblast origin. Blot is representative of n=4 experiments. **(B)** Western blot showing that both MEDIUM/LARGE STB-EVs and SMALL STB-EVs do not express APOA1. Plasma sample was used as a positive control for APOA1 expression. **(C)** Representative Western blot of Cytochrome C expression in whole placental lysates, MEDIUM/LARGE STB-EVs and SMALL STB-EVs isolated from control term placentae.

**Supplementary Figure 2S. Representative flow cytometry analysis of platelet-poor plasma (PPP) from peripheral and uterine vein.**

MEDIUM/LARGE STB-EVs were recovered from the top of the filter unit in 100 μl of filtered PBS, and stained with Bio-maleimide-FITC. The top panel (A, B, C) shows flow cytometry analysis of PPP plasma obtained from peripheral vein, while bottom panel shows analysis of uterine vein (D, E, F).

**A & D**) Filtrate was used to determine Bio-maleimide positive and the contaminating markers („Dump Channel‟) negative gate. „Dump Channel‟ gate was draw at 1% cut off using Dump channel-PeCy7 vs. Bio-maleimide-FITC dot plot. „Dump Channel‟ includes contaminant markers such as CD231a/b, CD41, HLA-ABC and HLA Class II; all conjugated with PeCy7 labelling.

**B & E**) In order to draw 1% cut off gates for anti-DPPIV-Alexa Fluor 647 and anti-PLAP-PE staining, samples were treated with detergent (NP-40) prior to events acquisition.

**C & F**) „Dump Channel‟ negative and Bio-maleimide positive population was analysed for PLAP and DPPIV double positivity.

**Supplementary Figure 3S. Specificity of PLAP antibody confirmed by western blotting using small extracellular vesicles (SMALL EVs) from different sources.**

PLAP antibody (NDOG2) showed no reactivity with small EVs isolated from MDA-MB-231, EAHY923, HUVEC or HEK293T Cells, as well as no reactivity with HepG2 Cells. Whole placental lysate, MEDIUM/LARGE STB-EV and SMALL STB-EV isolated from control term placentae were used as a positive control for PLAP antibody. Syntenin was used as a loading control for small EVs.
