# Supplementary material for: Placental extracellular vesicles express active dipeptidyl peptidase IV; levels are increased in gestational diabetes mellitus
Source: J Extracell Vesicles. 2019 May 23;8(1):1617000. doi: 10.1080/20013078.2019.1617000 (PMC6534242; doi:10.1080/20013078.2019.1617000)
Supplement: Supplemental Material [file ZJEV_A_1617000_SM1600.zip › Supplementary_Figures_after_revision_DPPIV.pptx]

## Slide 1
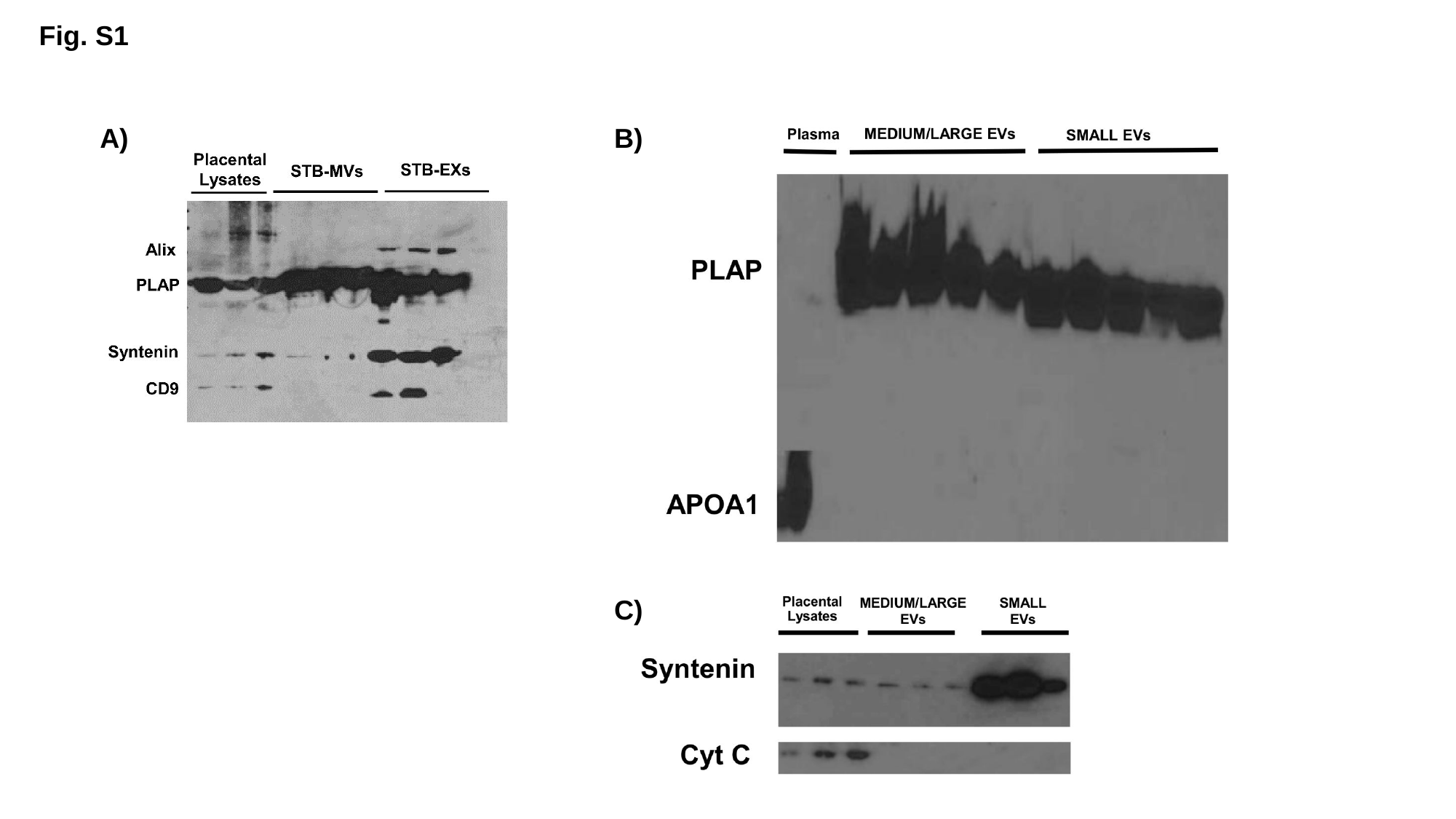

Fig. S1
A)
B)
C)

## Slide 2
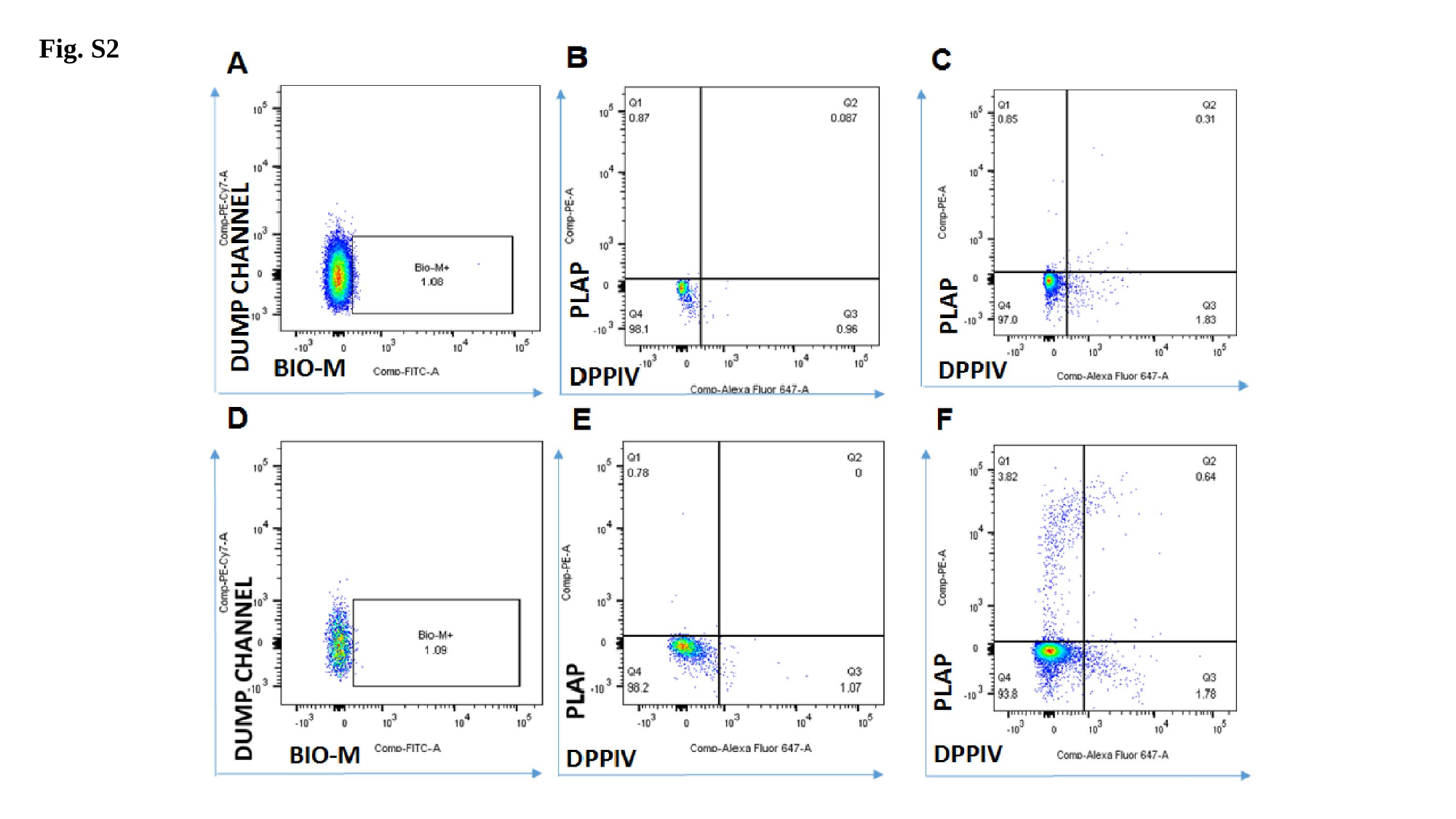

Fig. S2

## Slide 3
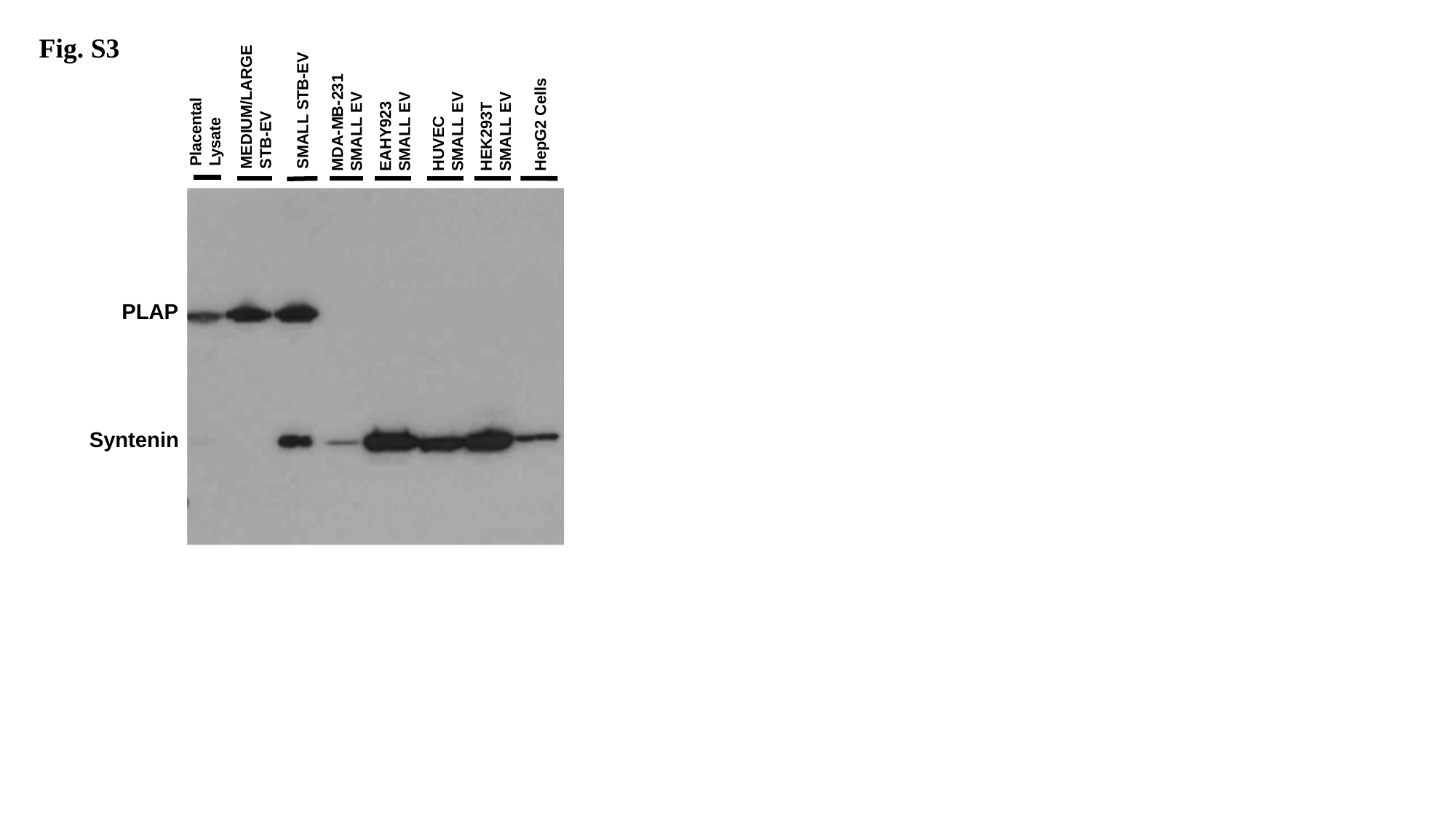

Fig. S3
MEDIUM/LARGE STB-EV
MDA-MB-231 SMALL EV
EAHY923
SMALL EV
HUVEC
SMALL EV
HEK293T
SMALL EV
SMALL STB-EV
Placental Lysate
HepG2 Cells
PLAP
Syntenin
